# Does the provision of a DVD-based audio-visual presentation improve recruitment in a clinical trial? A randomised trial of DVD trial invitations

**DOI:** 10.1186/s12874-019-0663-6

**Published:** 2019-01-30

**Authors:** Amy Rogers, Robert W. V. Flynn, Isla S. Mackenzie, Thomas M. MacDonald

**Affiliations:** Medicines Monitoring Unit (MEMO) and Hypertension Research Centre Division of Cardiovascular and Diabetes Medicine, University of Dundee Ninewells Hospital and Medical School, Dundee, United Kingdom

**Keywords:** Clinical trials, Recruitment, Informed consent, DVD, Audiovisual aids

## Abstract

**Background:**

Recruitment to clinical trials can be challenging. Methods that improve the efficiency of trial recruitment are needed to increase successful study completions. The aim of this study was to ascertain whether sending an audio-visual presentation on a digital versatile disc (DVD), along with usual study invitation materials, would improve recruitment to the Febuxostat versus Allopurinol Streamlined Trial (FAST), a clinical trial in patients with established gout.

**Methods:**

Potential participants for the FAST study who were identified by searches of GP records in Scottish primary care practices between August 2013 and July 2014 were included in this study. Individuals were randomly allocated to receive either a standard invitation (letter and information leaflet) or a standard invitation and a DVD containing an audio-visual presentation explaining the background and operation of FAST. Data on invitation response rates, screening attendances and randomisations were collected by research nurses.

**Results:**

One thousand fifty potential participants were invited to take part in FAST during this period. 509 individuals were randomised to receive the DVD presentation and the standard invitation and 541 received a standard invitation only.

DVD recipients were less likely to respond to the initial invitation (adjusted OR 0.76, CI 0.58–0.99) and marginally less likely to return a positive response (OR 0.75, CI 0.59–0.96). There was no statistically significant difference between the groups in attendance for screening or randomisation. The DVD did not influence the age, gender, or socioeconomic deprivation scores of those responding positively to a letter of invitation.

**Conclusions:**

The inclusion of a DVD presentation with FAST study invitations did not make any practical difference to the rate of positive response to invitation. Further innovation and evaluation will be required to improve recruitment to clinical trials.

**Trial registration:**

EU Clinical Trials Register. EudraCT Number: 2011–001883-23.

ISRCTN registry. ISRCTN72443278.

**Electronic supplementary material:**

The online version of this article (10.1186/s12874-019-0663-6) contains supplementary material, which is available to authorized users.

## Background

Recruitment of participants to clinical trials is a major challenge for investigators. When trials fail to recruit adequate numbers of participants this can result in underpowered studies that fail to address their research questions. It has been estimated that less than half of studies achieve pre-specified recruitment targets with implications for the duration and feasibility of studies and their statistical power [1]. Effective measures to improve recruitment rates would have a positive effect on the number of studies reporting meaningful results in a timely manner. A number of interventions have been described that aimed to improve the recruitment process including alterations to study design, recruitment methods, and consent process and incentives for participants. Of these, only telephone reminders, in studies with low baseline recruitment, have been shown to be effective in increasing recruitment. For the rest, there remains a lack of good quality, generalisable evidence about which of these diverse interventions actually work [1, 2].

As long ago as 1978, video was being used to impart information to potential trial participants [3]. Recent advances in the cost and availability of audio-visual recording and viewing equipment have made this approach more viable for large scale trials. This has led some researchers to investigate whether these modalities could enhance trial recruitment [4, 5]. A Cochrane review, by Synnot et al., in 2014 concluded that, while there was limited low-quality evidence that audio-visual aids may improve potential participants’ knowledge and understanding of a trial, they had been found to make little difference to eventual participation rates [6]. A more recent Cochrane review, updated in 2018 by Treweek and colleagues, also found only limited low quality evidence on the effect of providing audio-visual materials in addition to standard consent materials on rates of participation or intent to participate [1]. All 3 studies included in this more recent review tested a generic video about clinical trial participation rather than a presentation about a specific trial. Also, only one of those tested the effect of the video in a population who were eligible to take part in a clinical trial [4].

This study aimed to test the hypothesis that a study-specific DVD-based audio-visual presentation sent to potential study participants alongside the usual written materials would improve rates of randomisation in comparison to usual study invitation materials alone.

The FAST study is a large scale prospective, randomised, open-label, blinded endpoint clinical trial designed to evaluate the long term cardiovascular safety of febuxostat in comparison with allopurinol in the treatment of gout [7]. The study aims to randomise 5706 participants to either febuxostat or allopurinol. In a study of this size recruitment is often a lengthy and expensive process. Improvement to the efficiency of trial recruitment could lead to cost savings and shorter trial duration.

## Methods

### FAST study overview

The FAST study (EudraCT No: 2011–001883-23, ISRCTN72443278) is being conducted in Scotland, England and Denmark - this paper concerns only participants in Scotland recruited during a defined time period.

Potential participants for the FAST trial were identified by an automated search of participating GP (General Medical Practitioner) records. Suitable patients were aged 60 or over, taking allopurinol for chronic gout, and with additional cardiovascular risk factors. Letters and study information leaflets were sent by post to suitable patients from participating practices inviting them to arrange a visit with a research nurse to find out more about the study and to determine if they were eligible.

### Aim

The aim of this study was to ascertain whether sending an audio-visual presentation on a digital versatile disc (DVD), along with study invitation materials, would improve recruitment to the Febuxostat versus Allopurinol Streamlined Trial (FAST), a clinical trial in patients with established gout. The primary outcome was positive response to invitation. Secondary outcomes included any response to invitation, attendance for screening, and eventual randomisation. We also explored the effect of the DVD on the demographic profile of randomised participants and the cost-effectiveness of the intervention.

### Design and setting

In this randomised parallel group sub-study potential FAST study participants in 58 participating primary care practices in the UK were randomly assigned to receive a DVD presentation in addition to the usual written materials or standard invitations alone. Randomization was done centrally at the Medicines Monitoring Unit, Dundee (trial coordinating centre), on a 1:1 basis, using randomly generated numbers. A formal prospective sample size calculation was not performed for this study.

The usual FAST study invitation packs comprise a patient information sheet, a sample FAST study consent form, an invitation letter and a patient response form. These are sent by second class mail from the recruiting practices. The DVD invitation packs contained the same paper components with the addition of a DVD packaged in a slim plastic clamshell.

The DVD presentation was prepared by the study team with assistance from a video production company. The resulting presentation was approved by the local research ethics committee. The DVD contained footage of a professional actor playing the part of King Henry VIII of England, a well-known sufferer of gout. It also contained footage from members of the FAST study team explaining the various aspects of the trial. The full footage is available to view online at https://youtu.be/uUSBHoaQzI8.

**Fig. 1:**
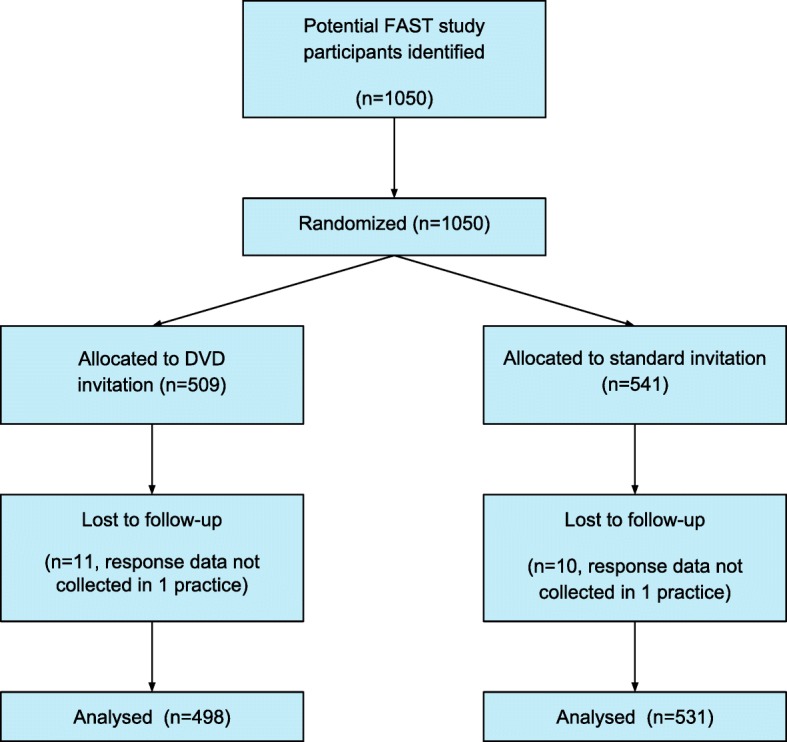
FAST DVD study CONSORT flow diagram

Those individuals responding positively to an invitation were contacted by a study nurse and invited to attend an information and screening appointment. Suitable and consenting participants were then randomised within the FAST study. Study nurses were not blinded to the DVD group allocation since the primary endpoint of positive response to invitation occurred prior to screening visits. A CONSORT study flow diagram is shown in Fig. 1 (a CONSORT checklist is also available in the Additional files 1 and 2).

Routinely collected demographic and response data, including age, gender and postcode were recorded for each participant. The Scottish Index of Multiple Deprivation (SIMD) 2012 was used to estimate socioeconomic deprivation. The SIMD is a postcode based measure of socioeconomic status taking into account seven contributing domains: income, housing, health, education, employment, skills, and crime [8]. The first portion of participant postcodes were used to calculate a median SIMD for the area that they live in.

### Statistical analysis

Chi-squared testing (for categorical variables) and t-tests (for continuous variables) were used to determine significant differences in outcome between the two (DVD and non-DVD) groups. Logistic regression modelling was used to examine the effect of potential co-variants affecting positive response rate. All analyses were performed on anonymised data using SAS version 9.4 software (SAS Institute, Cary, NC, USA).

## Results

Between August 2013 and July 2014, 58 primary care practices agreed to invite their patients to take part in the FAST study. The median number of invitations sent per practice was 14 (range 1–95). A total of 1050 potential participants were identified and invited to take part in FAST. 509 individuals received the DVD presentation and 541 a standard invitation only. As follow-up response data was not submitted for one practice, the affected 18 individuals were removed from the analysis. The remaining 501 individuals who received the DVD and 531 who did not were included in the analysis. The mean age of invited participants was 70.9 ± 7.0 years, and 80.2% were male. The two groups were balanced for age, gender, and deprivation, as measured by the postcode based Scottish Index of Multiple Deprivation (SIMD). The participant characteristics are summarised in Table 1 [8].

**Table 1:** Baseline characteristics of the sample

Characteristics		Total (*n* = 1050)		DVD (*n* = 501)		no-DVD (*n* = 531)
	Mean(SD)	*n*(%)	Mean(SD)	*n*(%)	Mean(SD)	*n*(%)
Age (years)	70.9 (7.0)		70.8 (7.0)		71.0 (7.0)	
Sex						
Male		828 (80.2)		403 (80.4)		425 (80.0)
Female		204 (19.8)		98 (19.6)		106 (20.0)
SIMD quintile						
1 (most deprived)		49 (4.8)		26 (5.2)		23 (4.3)
2		192 (18.6)		86 (17.2)		106 (20.0)
3		243 (23.6)		119 (23.8)		124 (23.4)
4		382 (37.0)		190 (37.9)		192 (36.2)
5 (least deprived)		166 (16.1)		80 (16.0)		86 (16.2)

### Primary outcome: Positive response to invitation

Five hundred seventy-nine responses by postage-paid reply slip were received within 6 weeks of invitations being sent out (56.1%). 321 of these recipients (31.1%) indicated that they would be willing to attend a screening appointment.

There was a marginal statistically significant difference in positive response rate (5.8%) between the two groups with DVD recipients being less likely to respond positively (absolute effect − 7, odds ratio (OR) 0.76, 95% confidence interval (CI) 0.59–0.995, *p* = 0.04). A logistic regression model including DVD, age, gender and deprivation (SIMD quintile) demonstrated that DVD, gender and deprivation level were significantly associated with positive response to invitation. There were no significant interactions between these variables. After adjusting for co-variants, DVD recipients remained less likely to respond positively than those receiving the standard invitation (adjusted OR 0.76, CI 0.58–0.99). Females were less likely to respond positively than males (adjusted OR 0.59, CI 0.41–0.85). Respondents from areas in SIMD 4th quintile were more likely to wish to take part compared to those in the middle SIMD 3rd quintile (adjusted OR 1.61, CI 1.13–2.29).

### Secondary outcomes

The outcomes of invitations for DVD and non-DVD recipients are displayed in Table 2.

**Table 2:** Invitation outcomes

Outcome	Total (*n* = 1032) *n* (%)	DVD (*n* = 501) *n* (%)	no-DVD (*n* = 531) *n* (%)
Responded to invitation	579 (56.1)	263 (52.5)	316 (59.5)
Responded positively	321 (31.1)	141 (28.1)	180 (33.9)
Attended for Screening	267 (25.9)	124 (24.8)	143 (26.9)
Randomised	243 (23.5)	114 (22.8)	129 (24.3)

There was a statistically significant difference between the two groups in terms of responding at all to the invitation, positively or negatively, with DVD recipients less likely to respond (OR 0.75, CI 0.59–0.96). The DVD made no statistically significant difference to whether an individual attended for a screening appointment or was randomised.

### Effect of DVD on demographics of positive responders

The DVD was not associated with any significant difference in the age, gender or deprivation of those responding positively to invitation. The demographics of randomised participants are shown in Table 3.

**Table 3:** Characteristics of randomised participants with versus without DVD

Characteristics		DVD (*n* = 114)		no-DVD (*n* = 129)
	Mean(SD)	*n*(%)	Mean(SD)	*n*(%)
Age (years)	70.1 (5.8)		70.6 (6.3)	
Sex				
Male		98 (86.0)		114 (88.4)
Female		16 (14.0)		15 (11.6)
SIMD quintile				
1 (most deprived)		1 (0.8)		4 (3.1)
2		19 (16.7)		22 (17.1)
3		19 (16.7)		24 (18.6)
4		58 (50.9)		57 (44.2)
5 (least deprived)		17 (14.9)		22 (17.1)

### Cost effectiveness

The additional cost of sending a DVD invitation pack in place of a standard invitation was £31.48, or £138.32 per randomised participant (see Table 4 for cost breakdown).

**Table 4:** Cost breakdown

Item	Project costs (£)	Additional cost per invitation sent (£)	Additional cost per randomised participant (£)
Video production	15,000	29.94	131.58
DVD manufacture and packaging	450	0.90	3.95
postage	318.72	0.64	2.79
TOTAL	15,768.72	31.48	138.32

## Discussion

This study did not find any benefit from including an audio-visual presentation along with usual recruitment materials. Of note, the overall response rate to letters of invitation was 56.6% with only 31.3% of recipients wishing to be contacted to arrange screening. This rate is similar to the observed response rate to invitations in other studies that have been coordinated by the same trial centre. It had been hoped that the addition of a DVD would increase uptake from areas of higher social deprivation; this was not demonstrated. Indeed, the DVD was associated with a reduced overall response rate and reduced positive response rate. One possible explanation for this is that the professionally produced DVD made the study appear more commercial in nature and less appealing to those inclined to take part in an academic study. Alternatively, lack of readily available DVD viewing equipment may have reduced engagement with the invitation on initial arrival and increased the chance of it being put to one side to be dealt with later and subsequently forgotten. No effort was made to ascertain if recipients actually watched the supplied DVD nor was any measurement of knowledge and understanding attempted. As such the mechanism of the observed effect of the DVD is unclear.

The FAST DVD presentation was a straightforward video presentation with no interactive or text features, other than optional English subtitles. Interactive computer-based information has been found to be increase willingness to take part in mock studies [9]. Advances in audio-visual recording and editing along with increasing public access to the internet open up further opportunities to develop more interactive forms of study information provision.

This sub-study is limited by its relatively small scale. It refers to recruitment in only a single study and was conducted only in Scotland. Potential participants in the FAST study are all aged 60 or over and sufferers of gout. This may limit the external validity of these findings and their usefulness to researchers planning studies with different or wider inclusion criteria. The SIMD quintiles used to estimate deprivation levels of participants were calculated using median SIMD for the first section of UK postcode only. This may have obscured significant street by street variation in deprivation.

Hutchison et al. suggested that the real challenge in trial recruitment is to reach those potential participants who have already decided that clinical trials are not for them [4]. The “Get Randomised” media campaign in Scotland was successful in raising awareness of research but was not able to demonstrate an associated increase in willingness to take part [10]. A societal shift in opinion may be what is required to facilitate improved recruitment to traditional randomised controlled trials. However, it should be remembered that better knowledge and understanding may, rightfully, result in lower overall trial participation as potential participants would be better equipped to make a truly informed choices in their own interests.

Alternatively, novel methodologies combining randomisation with observational methods could effectively bypass this individual opt-in consent stage that so limits current research [11]. Whether such approaches would be ethically acceptable in practice remains to be demonstrated.

The low rate of response to invitation to participate remains a problem for many studies and further innovative methods to improve this would be welcome. However, as suggested by Treweek et al., researchers should perhaps focus efforts first on improving the evidence base for existing interventions in a coordinated manner to reduce avoidable research waste and maximise usable evidence. Engagement with initiatives such as the PRioRiTy project and www.trialforge.org should be encouraged [12]. Additionally, the complex and often situation-specific nature of trial recruitment implies that that future interventions may benefit from additional qualitative evaluation to improve development and implementation [13].

## Conclusions

This study demonstrated that sending a DVD explaining the FAST study along with a standard invitation was ineffective in increasing the positive response rate. There was no indication that inclusion of a DVD would affect the overall deprivation status (as judged by postcode), age or gender of participants. Not only was the DVD associated with a reduced response rate, but it added significantly to the costs of recruiting one randomised participant. Based on our experience we do not recommend this methodology as an aid to recruitment.

## Additional files

Additional file 1: CONSORT checklist. Completed CONSORT checklist. (DOCX 60 kb)

Additional file 2: Dataset. De-identified dataset used for analysis. (CSV 21 kb)

## Abbreviations

DVD: Digital versatile disc FAST Febuxostat versus Allopurinol Streamlined Trial GP General Medical Practitioner SIMD Scottish Index of Multiple Deprivation
